# A Theoretical and Practical Treatise on Midwifery Including the Diseases of Pregnancy and Parturition

**Published:** 1850-11

**Authors:** 


### ARTICLE II.

*A Theoretical and Practical Treatise on Midwifery, including the Diseases of Pregnancy and Parturition.*—By P. CAZEAU, Adjunct Professor in the Faculty of Medicine of Paris, etc., etc. (Adopted by the Royal Council of Instruction.) Translated from the second French edition with occasional notes and a copius index by ROBERT P. THOMAS, M.D., Member of the Philadelphia County Medical Society; late Demonstrator of Anatomy in the Franklin Medical College, etc. With one hundred and seventeen illustrations; pp. 765; 8vo. Philadelphia: Lindsay & Blakison, 1850.

This is a well digested treatise, translated in a clear and good style, and got up in the usual excellent manner of the publishers.

Although we have a number of text books, for students and practitioners upon the subject of obstetrics already before the profession, we think there is still room, especially as every new work should embody the improvements and discoveries that are constantly being made.

The French are, generally, well posted up in reference to improvements, and the work before us shows abundant evidence of the fact. E.

